# Evaluating the safety and efficacy of a novel polysaccharide hemostatic system during surgery: A multicenter multispecialty prospective randomized controlled trial^☆^^☆☆^

**DOI:** 10.1016/j.sopen.2024.04.009

**Published:** 2024-05-03

**Authors:** Michael G. House, Robin Kim, Elaine E. Tseng, Ronald P. Kaufman, Marc R. Moon, Adam Yopp, Viraj A. Master

**Affiliations:** a Indiana University, Indianapolis, IN, United States of America; b University of Utah Hospital, Salt Lake City, UT, United States of America; c San Francisco Veterans Affairs Healthcare System, San Francisco, CA; d Albany Medical College, Albany, NY, United States of America; e Baylor College of Medicine, Houston, TX, United States of America; f University of Texas Southwestern, Dallas, TX, United States of America; g Emory University, Atlanta, GA, United States of America

**Keywords:** Cardiac surgery, General surgery, Hemostasis, Hemostatic agent, Intraoperative bleeding polysaccharide, Postoperative bleeding, Urologic surgery

## Abstract

**Background:**

Operative blood loss is associated with postoperative morbidity and mortality in surgery. Hemostatic agents are used as adjuncts for hemostasis during surgery and help to prevent postoperative bleeding. We evaluated the safety and efficacy of an investigational polysaccharide hemostatic (PH) topical product compared to a U.S. Food and Drug Administration (FDA)-approved control in clinical use comprising microporous polysaccharide hemospheres (MPH) to achieve hemostasis of bleeding surfaces during surgery.

**Study design:**

This prospective multicenter trial enrolled patients undergoing open elective cardiac, general, or urologic surgery. Patients were stratified by bleeding severity and therapeutic area, then randomized 1:1 to receive PH or MPH. Bleeding assessments occurred intraoperatively using a novel bleeding assessment methodology. Primary endpoint was noninferiority as compared with control via effective hemostasis at 7 min. Patients were monitored and followed daily in the postoperative period until time of discharge and again at 6 weeks. Overall survival was assessed in oncology patients at 24 months. Safety of PH vs. MPH was determined by comparing relative incidence of adverse events.

**Results:**

Across 19 centers, 324 (161 PH, 163 MPH) patients were randomized (48 % general surgery, 27 % cardiac surgery, and 25 % urologic surgery). PH was noninferior to MPH and met the primary endpoint of hemostatic success at 7 min at a non-inferiority margin of 10 %. No significant differences were found in adverse event rates. Six deaths were reported within the 6-week follow-up period. No difference in overall survival was observed at 2 years (76 % PH vs. 74 % MPH, *P* = .66) for patients undergoing cancer operations.

**Conclusion:**

Across three therapeutic areas, PH was noninferior to MPH at all hemostasis assessment time points with no safety concerns. PH is an effective alternative to MPH for hemostasis during surgery.

ClinicalTrials.gov Identifier: NCT02359994

## Graphical abstract

**Figure:**
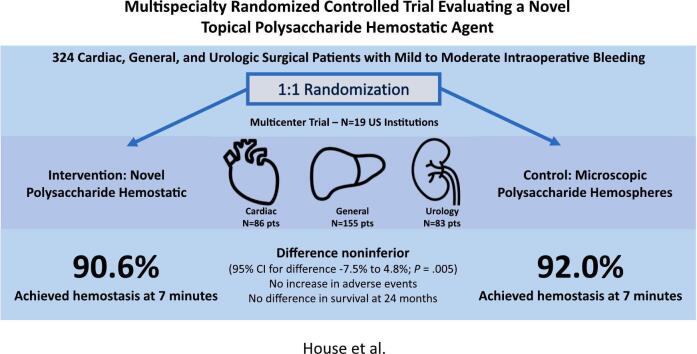

## Introduction

Blood loss related to surgical procedures is a critical factor in postoperative outcomes across numerous surgical specialties [1,2]. Operative blood loss and perioperative transfusions are associated with morbidity and mortality, including decreased survival, organ failure, infection, and longer hospital stays [[3], [4], [5], [6]]. Avoiding peri- and postoperative bleeding from organ and tissue surfaces is associated with optimal outcomes in surgery.

In addition to conventional methods of hemostasis such as vessel ligation and electrocautery, topical hemostatic agents have been developed and used during operative procedures [7,8]. Some of these agents contain clot-activating factors, while others aid in hemostasis by hemoconcentration and plug formation. Hemostatic agents that do not contain human- or animal-derived products carry several advantages including no risk of human- or animal-borne infection transmission [9]. Such agents may improve hemostasis in various operative procedures and are particularly useful in clinical situations that must avoid blood transfusions [[10], [11], [12]].

A novel polysaccharide hemostatic (PH) agent derived from purified starch has been developed to aid hemostasis via rapid dehydration of blood, leading to increased concentration of blood cells and coagulation factors. This results in acceleration of intrinsic and extrinsic clotting pathways [13]. This product is intended to be administered topically in open or laparoscopic procedures to help achieve hemostasis after conventional techniques are attempted [13]. This study was designed to test the hypothesis that PH is noninferior to a commercial product currently in clinical use that consists of microporous polysaccharide hemospheres (MPH) [14].

## Methods

Participants were enrolled at 19 surgical centers in the United States that received institutional review board approval. Informed consent was obtained for all participants in accordance with Good Clinical Practice guidelines and the Declaration of Helsinki. This trial was performed under an Investigational Device Exemption granted by the U.S. Food and Drug Administration. This trial was registered on ClinicalTrials.gov with identifier NCT02359994.

### Study endpoints

This study was a prospective, multicenter, randomized controlled trial to determine noninferiority of PH compared with MPH. The primary endpoint was noninferiority of PH for successful hemostasis of persistent bleeding within 7 min of application of topical hemostatic agent after conventional methods of hemostasis had been employed. Secondary endpoints included hemostasis within 5 min of application of hemostatic agent and safety outcomes.

### Eligibility criteria

Participants were assessed for eligibility preoperatively and intraoperatively. Preoperative assessment was determined within 30 days prior to surgery. Only patients who met preoperative and intraoperative suitability were included in the analysis. Preoperative inclusion criteria were age greater than 22 and able and willing to give informed consent for participation. Participants needed to be undergoing an elective open procedure of a prespecified general, cardiac, or urologic type. Qualified cardiac procedures involved the epicardium, aortic anastomosis, or aortotomy suture lines. General surgery procedures comprised liver resection or total splenectomy. Urologic procedures included open on-clamp partial nephrectomy or open radical nephrectomy.

Preoperative exclusion criteria included known sensitivity to starch or starch-derived material; clinically significant coagulation disorder or disease defined as a platelet count of less than 100,000 cells per μL, international normalized ratio greater than 1.5, or partial thromboplastin time more than 1.5 s outside of the laboratory's normal reference range; use of corticosteroids (excluding inhaled, topical ophthalmologic, or topical dermatologic corticosteroids) within 6 weeks prior to surgery; treatment with an investigational product without completion of the entire follow-up period for that investigational product; pregnancy, plan for pregnancy during the follow-up period, breast feeding; or serum hemoglobin A1c level greater than 9 %. Subjects who received platelet glycoprotein IIb/IIIa antagonist therapy less than 48 h prior to surgery were excluded. Minor changes to eligibility criteria were made early in the study enrollment period to increase compliance, which included removing a complement C3 blood test as an exclusion criterion, changing hepatic dysfunction to coagulopathy as an exclusion criterion, and removal of renal dysfunction as an exclusion criterion. The site coordinator and principal investigator enrolled participants and ensured that inclusion and exclusion criteria were met.

Participants who met preoperative inclusion criteria were assessed for intraoperative inclusion and exclusion criteria. Intraoperative inclusion criteria included undergoing one of the above specified procedures in which there was ongoing bleeding at the specified area for each surgical procedure after all visible vessels, suture holes, or suture line gaps greater than or equal to 2 mm in diameter were ligated, and any applicable methods for hemostasis were attempted. The first-line methods of controlling bleeding could include: pressure tamponade; vessel ligation with suture, clips, staples, electrocautery, argon beam coagulation; or reversal of anti-coagulation, as applicable. The intraoperative protocol specified an anatomic site of bleeding that was less than or equal to 25cm^2^; application site of hemostatic agent that was less than 47cm^2^; and bleeding flux (rate of blood flow) from the identified site that was greater than 0.000040 g per cm^2^ per second (ooze of blood) and less than or equal to 0.013 g per cm^2^ per second (slight bleeding).

Bleeding flux was determined by application of pre-weighed gauze to the site of bleeding with gentle pressure until blood was visible on the top layer or for 10 s. The blood-stained gauze was then weighed to determine the blood loss over the given area and time interval. Intraoperative exclusion criteria included major intraoperative bleeding as defined by Advanced Trauma Life Support hemorrhage class of II, III, or IV; active or potential infection at the surgical site; wound classification of CO (contaminated) or D (dirty or infected) based on the Centers for Disease Control and Prevention wound classification system.

### Study protocol

Participants who met preoperative and intraoperative eligibility criteria where then randomized in a 1:1 allocation to receive PH or MPH via envelope randomization. Randomization was stratified based on surgery type (cardiac, general, or urologic) and bleeding severity to ensure randomization was balanced between the two arms within each stratum. Each site had its own randomization envelopes for each bleeding score and therapeutic area, independent of other sites. The site coordinator executed randomization once intraoperative inclusion criteria were met. A random permuted block method for randomization was used. This was a single-blinded trial where subjects were blinded to the hemostatic treatment they received.

The treatment allocation was administered via the instructions for use of the respective device. For subjects receiving PH or MPH, the investigator was allowed to use up to two 5 g bellows of product per subject. The exact amount of administer product was determined by weight of used product. The site of bleeding was thoroughly covered with product. Gentle manual pressure with dry gauze was applied for 5 min, after which the site was assessed for hemostasis. Manual pressure with dry gauze was reapplied until the 7-min evaluation was performed. If hemostasis was achieved, the surgeon continued to monitor for 5 additional minutes to assess for maintenance of hemostasis. Hemostasis was considered successful if the surgeon observed complete secession of bleeding. Any bleeding egressing from the applied product at the evaluation time points was considered failure of hemostasis. If hemostasis was not achieved, the surgeon used alternative means to control bleeding.

### Safety monitoring

Participants were monitored daily during the in-hospital postoperative period and at a 6-week postoperative evaluation visit. For participants with an oncologic diagnosis, a 24-month postoperative evaluation was performed. Study participants were blinded to treatment allocation until the 6-week follow-up appointment.

### Statistical analysis

Subjects were randomized in a 1:1 ratio to PH or MPH with randomization stratified by bleeding severity and therapeutic area (general, cardiac, urology). Assuming a hemostasis success rate of 90 % for both the experimental and control groups and a noninferiority margin of 10 %, a sample size of 308 participants was deemed adequate to detect a difference in effect with a power of at least 80 %. Sample size was increased to 324 to account for a possible 5 % loss in either group.

Statistical analysis was performed to assess for noninferiority. The intervention would be considered noninferior if the lower bound of a 95 % confidence interval (CI) for the difference between the percentage of successful hemostasis with MPH and PH was greater than −10 %. A Farrington-Manning score test was used for hypothesis testing. Due to the study consisting of a noninferiority design, study success was based on an as-treated analysis. Intent-to-treat and per-protocol analyses were also performed. A poolability analysis was performed across surgical specialties for the primary outcome using an interaction *P*-value threshold of 0.15. If therapeutic area/treatment interaction had a *p*-value ≤.15 in the poolability analysis, then the results of the primary analysis would be reported from the subgroup adjusted logistic regression.

## Results

**Fig. 1:**
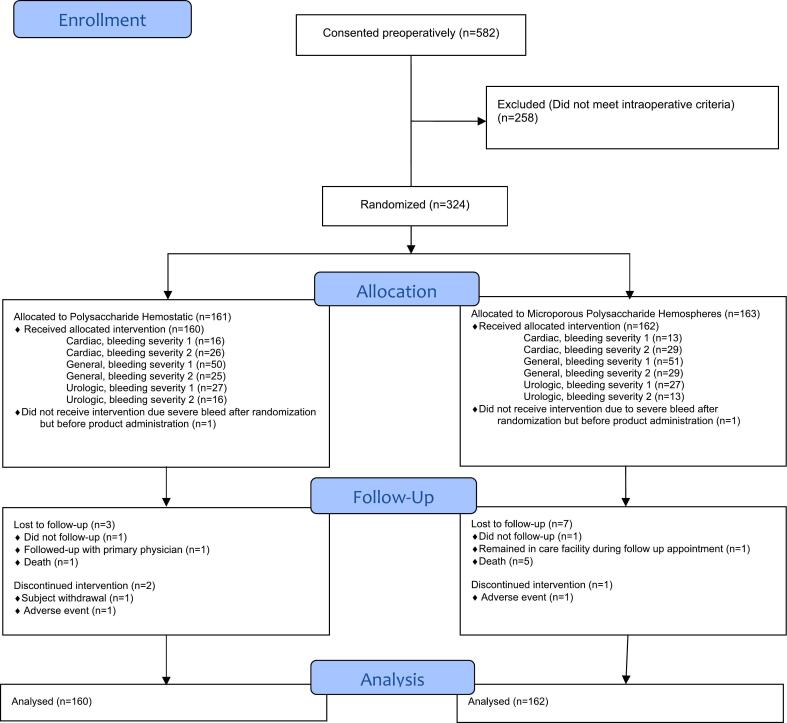
Flow diagram of participant participation.

From April 2015 to January 2019, 582 participants were consented. Of these, 324 participants met the preoperative and intraoperative inclusion criteria and were randomized. No major changes were made to the study protocol after initiation of enrollment. Baseline characteristics between the PH and MPH groups were similar, summarized in Table 1. A total of 160 participants in the PH group and 162 participants in the MPH group received the intervention per their randomization assignment. Study participants were included in the analysis of the primary and secondary endpoints (Fig. 1). Two major protocol violations involved participants randomized to receive a hemostatic agent who did not receive the assigned intervention; 1 for severe bleeding after randomization but prior to hemostatic application and 1 for breakthrough bleeding requiring cardiopulmonary bypass. Treatment groups did not cross over. The trial was ended when study recruitment requirements were met.

**Table 1:** Comparison of baseline characteristics of participants.

	Intervention	
	Polysaccharide Hemostatic (*n* = 161)	Microporous Polysaccharide Hemospheres (*n* = 163)
General characteristics		
Age, mean (SD), years	59.1 (13.76)	59.2 (13.85)
Height, mean (SD), cm	173.3 (10.09)	172.7 (9.69)
Weight, mean (SD), kg	86.0 (19.90)	87.0 (20.44)

Vital signs		
Systolic blood pressure, mean (SD), mmHg	135 (19)	131 (21)
Diastolic blood pressure, mean (SD), mmHg	78 (12)	75 (12)
Temperature, mean (SD) °C	36.6 (0.42)	36.6 (0.38)

Sex, n (%)		
Male	105 (65.2)	98 (60.1)
Female	56 (34.8)	65 (39.9)

Ethnicity, n (%)		
Hispanic or Latino	12 (7.5)	9 (5.5)
Not Hispanic or Latino	148 (91.9)	154 (94.5)
Unknown or not reported	1 (0.6)	0 (0)

Race, n (%)		
Asian	5 (3.1)	2 (1.2)
Black or African American	7 (4.3)	15 (9.2)
White	147 (91.3)	141 (86.5)
Other	2 (1.2)	5 (3.1)

Bleeding Severity Score,^a^ n (%)		
1	93 (57.8)	92 (56.4)
2	68 (42.2)	71 (43.6)

Comorbidity, n (%)		
Type 1 diabetes	2 (1.2)	1 (0.6)
Type 2 diabetes	31 (19.3)	36 (22.1)
Surgery type, n (%)		
Cardiac	43 (26.7)	43 (26.4)
General	75 (46.6)	80 (49.1)
Urologic	43 (26.7)	40 (24.5)

Laboratory		
Hemoglobin, g/dL, mean, (SD)	13.2 (1.57)	13.1 (1.83)
Platelet Count, 1000/μL. median (range)	212 (101–682)	227 (70–577)
International normalized ratio, mean (SD)	1.0 (0.10)	1.0 (0.09)

a Bleeding severity score based on area of bleeding and bleeding rate of flow. 0 = no bleeding, 1 = ooze, 2 = slight bleeding, 3 = moderate bleeding, 4 = severe bleeding, and 5 = life-threatening bleeding. SD = standard deviation.

### Study endpoints

The primary endpoint of hemostasis control within 7 min was achieved in 90.6 % of the PH group and 92.0 % of the MPH group (absolute difference − 1.4 %; 95 % CI -7.5 % to 4.8 %; *P* = .005 for noninferiority). No differences in outcome were found with intention-to-treat and per-protocol analyses. The secondary endpoint of hemostasis in 5 min was achieved in 90.0 % of the PH group and 85.2 % of the MPH group (absolute difference 4.8 %; 95 % CI -2.4 % to 12.0 %; *P* < .001 for noninferiority), with similar results for intention-to-treat and per-protocol analyses. Not only are results important at 5 and 7 min, but also comparing hemostasis maintenance or lack of re-bleeds should factor into effectiveness evaluation. MPH demonstrated 6 cases (2 general and 4 urology) that did not maintain hemostasis at 12 min vs. only 1 case (general) for PH. A total of 90.0 % of the PH and 88.3 % of the MPH group had hemostasis maintained at 12 min (absolute difference 1.7 %; 95 % CI -5.1 % to 8.5 %). Percentage of those achieving hemostasis by 5 min, 7 min, and maintenance of hemostasis at 12 min is presented in Table 2.

**Table 2:** Hemostasis at 5, 7, and 12 min in the As treated analysis population.

Timepoint	PH %	MPH %	Difference PH – MPH (95 % CI)
5 min	90.0 %	85.2 %	4.8 % (−2.4 %, 12.0 %)
7 min	90.1 %	91.4 %	−1.3 % (−7.7 %, 5.0 %)
12 min	90.0 %	88.3 %	1.7 % (−5.1 %, 8.5 %)

A total of 156 participants in the PH group (96.9 %) and 155 participants in the MPH group (95.1 %) completed the protocolized clinical follow-up. Hemostasis at 7 min by therapeutic area and bleeding severity from an ad hoc analysis is presented in Table 3A. Separate reporting of hemostasis by therapeutic area and bleeding severity was pre-planned. An examination of the procedures performed in each therapeutic area was performed to assess whether specific procedures were primarily responsible for the results within a particular therapeutic area. The procedures within the general and urology therapeutic areas showed fairly consistent differences between PH and MPH. There was somewhat more variability within the cardiac therapeutic area. Hemostasis at 7 min for cardiac surgeries was 85.7 % for PH vs. 69.0 % for MPH. Poolability analysis revealed an interaction *P* value of 0.02. The results of the covariate-adjusted analysis of hemostasis at 7 min were 90.7 % for PH and 91.9 % for MPH (absolute difference 1.25 %; 95 % CI -7.1 % to 4.6 %). These results align with the pooled primary endpoint analysis at 7 min supporting the conclusion of non-inferiority between PH and MPH. Planned subgroup analyses based on bleeding severity, sex, and race did not reveal statistically significant differences between interventions. Table 3B compares the hemostatic success at 7 min for the two hemostatic agents according to specific procedure type and bleeding site.

**Table 3A:** Hemostasis at 7 min stratified by therapeutic area and bleeding severity.

Therapeutic Area	Bleeding Severity Score	PH % (n/N)	MPH % (n/N)
General	1	94.0 (47/50)	100.0 (51/51)
General	2	92.0 (23/25)	100.0 (29/29)
Cardiac	1	87.5 (14/16)	69.2 (9/13)
Cardiac	2	84.6 (22/26)	69.0 (20/29)
Urologic	1	92.6 (25/27)	100.0 (27/27)
Urologic	2	87.5 (14/16)	100.0 (13/13)

**Table 3B:** Hemostasis at 7 min according to procedure type and respective bleeding site.

Therapeutic Area	Procedure Type (Bleeding site)	PH % (n/N)	MPH % (n/N)	Difference PH-MPH (95 % CI)
General	Hepatectomy (liver surface)	93.2 % (69/74)	100.0 % (79/79)	−6.8 % (−12.4 %, −1.1 %)
General	Total Splenectomy (retroperitoneum)	100.0 % (1/1)	100.0 % (1/1)	0 % (NA, NA)
Cardiac	CABG (aortotomy closure site)	77.8 % (7/9)	82.4 % (14/17)	−4.6 % (−36.4 %, 27.3 %)
Cardiac	Valve Repair - Aortic and/or Mitral (epicardium)	88.9 % (16/18)	75.0 % (9/12)	13.9 % (−13.3 %, 41.1 %)
Cardiac	Other (aortic anastomosis)	86.7 % (13/15)	46.2 % (6/13)	40.5 % (5.8 %, 75.2 %)
Urology	On-clamp Partial Nephrectomy (kidney surface)	88.9 % (8/9)	100.0 % (10/10)	−11.1 % (−31.2 %, 9.0 %)
Urology	Radical Nephrectomy (retroperitoneum)	91.2 % (31/34)	100.0 % (30/30)	−8.8 % (−19.2 %, 1.6 %)

Safety endpoints for the PH group were no different than the MPH group. One death occurred in the PH group, and 5 deaths occurred in the MPH group. Serious adverse events occurred in 27 % of subjects with 69 occurring in the PH group and 83 occurring in the MPH group. Serious adverse events possibly related to the study device are listed in Table 4. For oncology participants in the PH group (*n* = 85), 65 participants completed the 2-year follow-up period with 19 (29 %) deaths and 1 participant lost to follow up. For oncology participants in the MPH group (*n* = 93), 72 participants completed the 2-year follow-up period with 21 (29 %) deaths. No significant difference was found in 2-year survival between the PH and MPH groups (76 % vs. 74 %; *P* = .66). Additional safety outcomes are summarized in Table 5.

**Table 4:** Serious adverse events possibly related to study interventions.

Event Category	Number of Subjects	
	Polysaccharide Hemostatic (n = 161)	Microporous Polysaccharide Hemospheres (n = 163)
Pleural effusion	1 (0.6 %)	0
Respiratory failure	1 (0.6 %)	0
Thromboembolic Event	1 (0.6 %)	0
Hepatic failure	0	1 (0.6 %)
Ileus	0	1 (0.6 %)
Sepsis	1 (0.6 %)	0
Abdominal infection	0	1 (0.6 %)
Distributive Shock	1 (0.6 %)	0
Duodenal perforation	0	1 (0.6 %)
Gastric perforation	1 (0.6 %)	0
Hematoma infection	1 (0.6 %)	0
Implant site fluid collection	1 (0.6 %)	0
Pericardial Tamponade	1 (0.6 %)	0
Perihepatic fluid collection	1 (0.6 %)	0
Pneumonia	1 (0.6 %)	0
Portal vein thrombosis	0	1 (0.6 %)
Uncontrolled Hemorrhage	0	1 (0.6 %)

**Table 5:** Additional safety outcomes in As-treated population.

	Polysaccharide Hemostatic (*n* = 160)	Microporous Polysaccharide Hemospheres (*n* = 162)
Total operative time, mean (SD), minutes	254.2 (126.40)	260.2 (123.13)
Hemostasis achieved by alternate means, %, (n/N)	10.0 (16/160)	10.5 (17/162)
Total estimated blood loss, mean (SD), mL	471.4 (476.91)	483.6 (609.38)
Total units blood transfused, mean (SD)	0.4 (0.93)	0.4 (0.95)
Reoperation,^a^ % (n/N)	10.6 (17/160)	6.2 (10/162)
Total hospitalization time, mean (SD), days	7.0 (6.58)	6.6 (5.29)

a None of the reoperations were due to bleeding at the product application site. SD = standard deviation.

## Discussion

This study was a noninferiority trial across multiple surgical specialties using an objective measure of blood loss that demonstrates noninferiority of PH compared with MPH for the primary endpoint of hemostasis at 7 min and the secondary endpoint of hemostasis at 5 min. There were no safety concerns identified. Previously, a retrospective study of PH use during head and neck surgery demonstrated that no patients had bleeding after their procedure with PH use [15]. The study was prospective and randomized in design, which further supports efficacy of PH for hemostasis during surgical procedures. When evaluating outcomes based on surgical subtype, PH had consistent performance across all surgical types while MPH had less effectiveness in cardiac procedures. Anecdotally, it was noted by multiple investigators that the PH appeared to form a gel on the epicardium that remained stable during cardiac contraction with less breakthrough bleeding. However, this study was not powered to detect differences between interventions for individual surgical subtypes. PH is bioinert, absorbable within 2 to 4 days of application, and is not derived from human or animal tissue, which are characteristics of an ideal hemostatic agent. These findings suggest PH is an effective option for control of hemorrhage after standard hemostasis techniques are employed.

Several published studies have assessed the effectiveness of MPH in cardiac and urologic procedures. A retrospective study of MPH use in cardiac procedures demonstrated reduction in hemostasis time, reduced postoperative chest tube output, reduced volume of packed red blood cell transfusions, and similar 30-day mortality [10]. Similarly, a case-series of live-donor nephrectomies demonstrated hemostasis in 100 % of participants with MPH use, without any identified postoperative complications [16]. Use of MPH during radical prostatectomies was associated with decreased drop in postoperative hemoglobin level [17]. Finally, use of MPH during sinus surgery resulted in less bleeding during these procedures. PH is differentiated from some hemostasis products because it is fully absorbed within 96 h. Some hemostasis devices, such as oxidized regenerated cellulose (ORC), take longer to resorb and may cause inflammation and delay wound healing due to its acidic nature [[18], [19], [20], [21],^23^]. ORC absorption depends upon amount used, degree of blood saturation and the tissue bed [18,19,22,23]. These all may lead to foreign body reactions causing granuloma formation due to residual cellulose presence after healing [[18], [19], [20], [21],^23^,^24^]. Additionally, unintended scattering of the ORC powder formulation may increase the risks of adhesions [2]. We have shown that topical hemostatic agents such as MPH and PH are noninferior and hence represent additional options for the control of hemorrhage during surgical procedures.

Postoperative hemorrhage is associated with increased risk of postoperative complications, increased rates of reoperation, longer intensive care unit stays, prolonged need for ventilatory support, increased need for blood transfusions, and increased cost [25]. Interventions that limit operative and post-operative hemorrhage may lead to decreased cost and improved outcomes [25]. Topical hemostatic agents such as PH represent an additional option for control of operative hemorrhage. This study demonstrates the potential for use of PH as a topical hemostatic agent across multiple surgical specialties. The versatility may be beneficial in terms of decreasing the number of unique products needed at medical facilities. Additionally, the novel method of measuring intraoperative bleeding employed in this study strengthens the conclusions of this trial.

One potential limitation to this study is the poolability of the results across surgical specialties. The study was not powered to determine noninferiority for each individual specialty area. Our analysis of the primary outcome across surgical specialties demonstrated that the variability in outcomes was largely due to better-than-expected performance of the MPH in general and urologic procedures and worse-than-expected performance in cardiac procedures. On secondary analysis, this was determined to be due to reduced efficacy of MPH for hemostasis in epicardial procedures, although no factor explaining this discrepancy could be determined. Across specialties, PH performed more consistently. Overall, these findings suggest that the consistent efficacy of PH across therapeutic areas and all evaluation time points (5, 7, and 12 min) provides evidence that PH is comparable with MPH with future use.

Major strengths of this study were the design and methodology. Bias was reduced as surgeons were blinded to the therapy allocation until the bleeding site was identified, stratified and randomization to a treatment group, which also created a homogenous distribution of product use in procedures. Investigators were not double blinded to treatment allocation and we recognize this could be considered a limitation of the study. Results of this study may not be germane to additional surgical procedures. However, the surgical procedures chosen were considered to be representative of procedures most commonly performed within each specialty, and the multicenter design supports relevance of results. The effectiveness of PH for more severe bleeding was not evaluated.

## Conclusion

The novel topical hemostatic PH was noninferior to MPH for successful hemostasis within 5 and 7 min for open general, cardiac, and urologic procedures. No safety concerns were identified. Our results demonstrate that PH is an effective alternative to MPH for controlling persistent bleeding after conventional methods of hemostasis are attempted.

## CRediT authorship contribution statement

**Michael G. House:** Writing – review & editing, Writing – original draft, Investigation, Formal analysis, Data curation. **Robin Kim:** Writing – review & editing, Writing – original draft, Investigation, Data curation. **Elaine E. Tseng:** Writing – review & editing, Writing – original draft, Investigation, Data curation. **Ronald P. Kaufman:** Writing – review & editing, Writing – original draft, Investigation. **Marc R. Moon:** Writing – review & editing, Writing – original draft, Investigation, Data curation. **Adam Yopp:** Writing – review & editing, Investigation, Data curation. **Viraj A. Master:** Writing – review & editing, Investigation.

## Declaration of competing interest

As the study sponsor, CryoLife/Artivion provided financial support to conduct the study including project management, monitoring, data management, statistical analyses, and generation of the final study report for submission to the FDA. The authors have disclosed any conflicts of interest.
